# Expression of CD39 on FoxP3^+ ^T regulatory cells correlates with progression of HBV infection

**DOI:** 10.1186/1471-2172-13-17

**Published:** 2012-04-11

**Authors:** Yan Tang, Li Jiang, Yanhua Zheng, Bing Ni, Yuzhang Wu

**Affiliations:** 1 Institute of Immunology, PLA, Third Military Medical University, Chongqing 400038, People Republic of China; 2 Institution of Infectious Diseases, Southwest Hospital, the Third Military Medical University of Chinese PLA, Chongqing 400038, People Republic of China; 3 Department of Pathology and Experimental Medicine, 306 Hospital of PLA, Beijing 100101, People Republic of China

**Keywords:** Hepatitis B, CD39, Regulatory T lymphocyte

## Abstract

**Background:**

Although it is known that regulatory T cells (Tregs) can suppress the function of effector T cells, and may contribute to impaired immune response, the precise role of Tregs during the course of hepatitis B virus (HBV) infection remains to be elucidated. A newly identified subset of the CD4^+^Foxp3^+ ^Tregs, the CD39^+ ^Tregs, has been associated with viral infections and autoimmune diseases. Therefore, we hypothesized that this discrete Treg subset may contribute to the chronic infection of HBV.

**Results:**

Initial characterization studies of healthy peripheral CD39^+^FoxP3^+^CD4^+ ^T cells revealed that the majority were CD45RA^- ^Treg cells. Subsequent analysis of HBV-infected patients (38 asymptomatic HBV carriers (AsCs), 37 chronic active hepatitis B (CAH), 29 HBV-associated acute-on-chronic liver failure (ACLF)) and healthy individuals (25 controls) was conducted to assess association with HBV copy number and the liver injury marker alanine aminotransferase (ALT). A higher percentage of CD39^+ ^Tregs was detected within the population of FoxP3^+^CD4^+ ^T cells in peripheral blood of AsCs patients. Moreover, the percentage of CD39^+ ^Tregs was significantly less in CAH and ACLF patients. The increased proportions of circulating CD39^+ ^Tregs were positively correlated with serum viral load, but inversely correlated with serum ALT level.

**Conclusion:**

These findings not only suggest that CD39^+ ^Treg cells may be involved in HBV disease progression but also identify CD39^+ ^Tregs as a dynamic immune regulatory cell population that may represent a new target of immunomodulatory therapeutic interventions.

## Background

Hepatitis B virus (HBV) is a non-cytopathic, hepatotropic DNA virus that is capable of inducing necro-inflammatory liver disease with varying severity. Persistent infection by HBV is often associated with chronic liver disease, which can further lead to the development of cirrhosis and hepatocellular carcinoma [1,2]. While many of the underlying mechanisms of HBV infection progression have been described, the complex array of pathogen-host interactions is not yet fully understood. A growing body of recent evidence has suggested that CD4^+^CD25^+^Foxp3^+ ^regulatory T cells (Tregs) may play an important role in the suppression of antiviral T cell responses during the chronic phases of HBV infection [3,4]. Some studies have demonstrated that HBV carriers have a higher frequency of Tregs in peripheral blood and liver than healthy controls or individuals with resolved infection, indicating that Tregs may contribute to HBV persistence [5-8]. However, other studies have failed to detect any differences in Treg frequencies between asymptomatic carriers (AsCs) and healthy controls [9]. Thus, the role of Tregs in the progression of liver disease remains controversial, and little is known about whether their potential roles in viral hepatitis pathogenesis differ according to their functional activities.

The largely discordant results among the studies that have assessed the role of Tregs in HBV infection may reflect variation in study design and model systems. For example, the definition of Tregs varied between studies, and in many was based solely upon the co-expression of CD4^+ ^and CD25^high^Foxp3^+^. Unlike the Tregs in mice, human Tregs are heterogeneous and the population exhibits considerable diversity. Phenotypically and functionally distinct subsets of Tregs can mediate immune suppression through distinct mechanisms, including specific profiles of immunomodulatory factors, such as IL-10, TGF-β, granzyme B, perforin, CTLA-4, GITR, and Lag-3 [10-12]. In addition, CD39-mediated interactions were recently implicated in Treg activities during different stages of viral infection [11,13-15].

CD39 is a recently described molecule with immunomodulatory properties, and is expressed on human and murine Tregs. Several studies have identified the CD39/NTPDase1 molecule as a useful biomarker of a CD4^+^FoxP3^+ ^regulatory suppressor T cell population with potent immunosuppressive activity in humans and mice [16,17]. Notably, a significant increase of CD39 expression on Tregs has been observed in cancer patients and patients with human immunodeficiency virus (HIV) infection, and a strong association was found between CD39 expression on Tregs and tumor or AIDS disease progression [13-15,18]. Decreased frequency and function of CD39^+ ^Tregs have been reported in multiple sclerosis [19], ryegrass allergy [20], and vascular inflammation after transplantation [21].

Although the critical roles of CD39 in some diseases have been described, the potential roles of CD39-expressing Treg cells in hepatitis B pathogenesis have yet to be elucidated. Therefore, this study was designed to investigate the phenotype characteristics and frequency of peripheral and intrahepatic Tregs and CD39^+ ^Tregs in hepatitis B patients, and to determine whether the characteristics of this subset are related to the HBV disease process.

## Methods

### Patients and healthy controls

Heparinized peripheral blood samples were obtained from 25 healthy individuals (controls) and 104 HBV-infected patients, including 38 AsCs, 37 chronic active hepatitis (CAH) and 29 HBV-associated acute-on-chronic liver failure (ACLF), all of whom were diagnosed according to the described criteria [22] (Table 1). Patients co-infected with HIV, hepatitis A virus, hepatitis C virus (HCV), or hepatitis D virus and patients with a resolved viral hepatitis (other than HBV) were excluded from this study. Patients and controls who were immunocompromised or pregnant and patients that received antiviral or immunomodulatory HBV treatment at any time during the six months prior to blood sampling were also excluded.

**Table 1:** Clinical characteristics of populations enrolled in the study

Group	HC	AsCs	CAH	ACLF
Cases	25	38	37	29
Sex, male/female	14/11	25/13	26/11	23/6
Age, years (range)	28 (18-35)	33 (20-67)	40 (18-62)	39 (23-61)
ALT, U/L	< 40	< 40	475 (50-2340)	429 (58-1885)
TBIL, μmol/L	ND	ND	95.6 (26-412)	439 (239-881)
PTA, %	ND	ND	94.1 (42.4-167)	34.8 (17.8-68.5)
HBVDNA, copies/mL (range)	ND	2.27 × 10^7^ (1.78 × 10^3^-1.51 × 10^8 ^)	2.68 × 10^7^ (0-5.07 × 10^8 ^)	3.58 × 10^7^ (0-4.64 × 10^8^)
HBsAg-positive	0	38	37	29
HBeAg-positive	0	21	15	12
HBeAb-positive	0	17	22	17
HBcAb-positive	0	38	37	29

ND: not determined.

Data are shown as median and range.

Liver samples were obtained by diagnostic liver needle-biopsy and immediately frozen in liquid nitrogen until analysis. HBV-infected liver tissue samples were obtained from distal liver tissue of the liver hemangioma patients with HBV infection. The degree of hepatic inflammation was graded using the modified histological activity index (HAI) described by Scheuer [23]. All samples were collected with the informed consent of the patients, and the experiments were approved by the ethics committee of the Third Military Medical University (Chongqing, China).

The clinical and immunological characteristics of the cohort are shown in Table 1.

### Flow cytometric (FCM) analysis and antibodies

The following antibodies were used to detect Treg subsets by FCM: anti-hCD4, anti-hCD3, anti-hCD45, anti-hCD25, anti-hCD45RA, anti-hCD39, anti-HLA-DR, anti-Ki-67, anti-FoxP3, anti-CTLA-4, and anti-IL-17A. All antibodies were purchased from eBioscience (San Diego, USA), except for anti-hCD39-FITC (Ancell, USA), anti-hCTLA-4 and -hIFN-γ (BD Biosciences, USA).

Peripheral blood mononuclear cells (PBMCs) or liver infiltrating lymphocytes (LILs) were obtained by Ficoll gradient and the cells were then stained following the protocol described in a previous study [6]. The usage of all antibodies for FCM staining followed the recommended condition by the manufactures. For detection of intracellular cytokines, CD4^+ ^T cells were stimulated with 50 ng/mL phorbol 12-myristate 13-acetate (PMA; Sigma-Aldrich, USA) and 1 μg/mL ionomycin (Sigma-Aldrich) in the presence of Golgi-Stop (BD Biosciences) for 5 hr, and then stained with anti-hIFN-γ or anti-hIL-17A after fixation and permeabilization. Immunoreactive data was acquired by a FACS CanTo II flow cytometer (BD Biosciences) and analyzed with Flowjo software (Tree Star Inc., Canada).

### Cell sorting

CD4^+^CD25^+ ^(> 90%) and CD4^+^CD25^- ^(> 90%) T cells were isolated from PBMCs of healthy controls by using the CD4^+^CD25^+ ^regulatory T cell isolation kit (Miltenyi Biotec, USA), according to the manufacturer's instructions. CD4^+^CD25^+ ^T cells were then stained with CD39-APC, CD25-PE, and CD4-PerCP-Cy5.5, respectively. Finally, CD4^+^CD25^+^CD39^+ ^and CD4^+^CD25^+^CD39^- ^Treg cell subsets were sorted using a FACSAria flow cytometer (BD Biosciences).

### Suppression assay

FACS-sorted CD4^+^CD25^+^CD39^+ ^or CD4^+^CD25^+^CD39^- ^"suppressor" T cells were first cocultured in triplicate with autologous CD4^+^CD25^- ^"responders" (10,0000 cells/well) in 96-well plates at the indicated suppressor/responder (S/R) ratios for three days with media alone, or 1 μg/mL plate-bound anti-CD3 (clone UCHT1; BD Pharmingen) with 2 μg/mL anti-CD28 (clone CD28.2; BD Pharmingen). Then, the cells were cultured for 16 h with [^3^H] thymidine (1 Ci/well) [24]. After harvesting the cell cultures and plating onto glass fiber filters, the radioactivity that had incorporated into the cells' DNA was measured as an indicator of cell proliferation by using a liquid scintillation counter (LS 6500; Beckman Coulter, Inc., USA).

### Immunohistochemistry analysis

Fresh liver biopsy specimens were embedded in the OCT compound (Tissue Tek, Japan) and stored at -80°C until use. Sections were then incubated with anti-hCD39 (1:50; eBioscience) or anti-hFoxP3 (1:50; eBioscience). After washing, sections were incubated with a secondary polymeric, peroxidase-labeled rabbit anti-mouse antibody (EnVision™ System; Dako, Denmark) for 30 min. Sections that were incubated with isotype- and concentration-matched immunoglobulin only, without primary antibodies, were used as isotype controls. Reactivity was detected with a DAB Elite kit (K3465; Dako) and brown coloration of tissues represented positive staining.

Immunofluorescence staining for human CD4/FoxP3/CD39 was performed as previously described [6]. Briefly, sections were incubated with anti-CD39 (1:200; eBioscience), anti-CD4 (1:100; Santa Cruz Biotechnologies, USA), or anti-FoxP3 (1:200; Abcam, UK), followed by incubation with Cy3-labeled anti-mouse IgG (1:500), Cy5-labeled anti-goat IgG (1:500), and Dylight488-labeled anti-rat IgG (1:200; Zhongshan Goldenbridge Biotechnology, China). Sections that had been incubated with the related isotype control antibodies and fluorescence-labeled secondary antibodies were used as isotype controls. Results were analyzed by fluorescence microscopy (Axioplan 2; Zeiss, Germany) and the positively stained cells were counted in a high-power field (hpf, ×400), according to the described protocols [8].

### Virological and immunological assessment

The levels of HBsAg, anti-HBs, anti-HBc, HBeAg, anti-HBe, anti-HCV, anti-HDV, anti-HGV, and anti-HIV were measured using commercially available kits (Abbot Laboratories, USA). HBV-DNA levels were determined using real-time polymerase chain reaction (Amplicor, Roche), according to the manufacturer's instructions. The threshold of the HBV DNA detection limit was 100 IU/mL.

### Statistical analysis

Data analysis was conducted with Prism 5.0 software (GraphPad Software, San Diego, CA) and R [http://www.R-project.org/]. When data can not follow Gaussian distribution, Kruskal-Wallis test was selected for overall comparisons, and post-hoc analysis was performed using the Steel-Dwass test for multiple comparisons among the groups. Meanwhile, data distributed as Gaussian distribution were shown as mean value ± standard deviation (SD), followed by student's *t *test for 2 independent samples or analysis of one way variance (ANOVA). Correlation analysis was carried out with Spearman rank correlation test. Presented *P *values were deemed significant at or below a 5% level.

## Results

### CD39 defines a discrete subset of CD4^+^Foxp3^+ ^T cells

**Figure 1:**
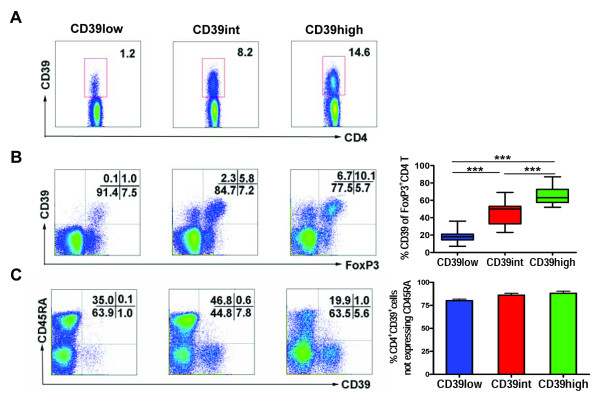
**CD39 was expressed variably but preferentially on memory Treg cells**. PBMCs from healthy donors were stained with anti-CD4, -CD39, -CD45RA, or -FoxP3 monoclonal antibodies (mAbs) and analyzed by FACS. Dot plots gated on CD4^+ ^T cells are shown from a representative sample. **(A) **Expression of CD39 in the gated CD4^+ ^T cells of PBMCs. **(B) **Co-expression of CD39 and FoxP3 in the gated CD4^+ ^T cells of PBMCs. Bar graph for **(B) **shows the percentage of CD39 expression on FoxP3^+^CD4^+ ^T cells. **(C) **Co-expression of CD39 and CD45RA in the gated CD4^+ ^T cells of PBMCs. The bar graph for **(C) **shows the percentage of CD39 expression on CD45RA^-^CD4^+ ^T cells. *** *P *< 0.001.

Recent data have shown that mouse Tregs constitutively express CD39 molecules [25], while the proportions of CD39^+ ^cells appear highly variable in humans [17,26]. Accordingly, in this study, FCM analysis revealed a high variance in the frequency of CD4^+ ^T cells expressing CD39, which ranged from 1% to 20% among the healthy donors and HBV-infected subjects (Additional file 1). Based on the distribution shown in the scatter diagram for the frequency of CD4^+ ^T cells expressing CD39, we subdivided the total cohort into three groups according the frequency of CD39-expressing CD4^+ ^T cells: CD39low (< 5%), CD39int (5 ~ 10%) and CD39high (> 10%), in order to characterize the CD39 expression with disease status in more detail (Figure 1A and Additional file 1).

We then stained these cells to detect intracellular FoxP3 expression and identified four CD4^+ ^T subsets in each group: CD39^+^Foxp3^+^, CD39^-^Foxp3^+^, CD39^+^Foxp3^- ^and CD39^-^Foxp3^- ^T cells (Figure 1B). We consistently observed an increasing trend of the proportion of CD39^+^Foxp3^+ ^Tregs in total Foxp3^+ ^Tregs in the CD39low (17%), CD39int (45%) and CD39high (65%) groups (Figure 1B), in accordance with the hypothesis that the CD39 surface marker could be used for routine isolation of functional human Tregs [27]. Since CD39 expression denotes memory CD4^+ ^T cells [28,29], we theorized that the highly variable expression of CD39 may affect the memory phenotype of CD4^+ ^T cells. Thus, we analyzed the CD45RA expression on CD39^+^CD4^+ ^T cells. The results showed that the majority of CD39^+^CD4^+ ^T cells were CD45RA^-^. Specifically, > 70% proportions of CD39^+^CD4^+ ^T cells (denoting memory phenotype) were found in each of the CD39low, CD39int and CD39high groups (Figure 1C).

**Figure 2:**
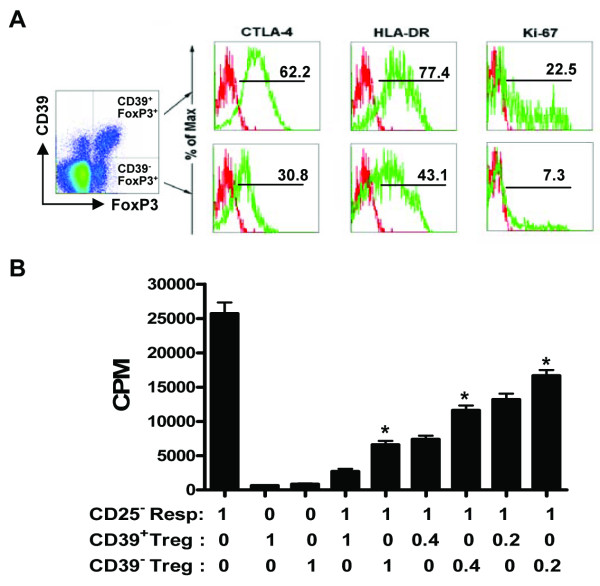
**CD39^+ ^Treg cells display strong suppressive effects and highly express the functional maker**. **(A) **The percentage of CTLA-4, HLA-DR and Ki-67 expression on CD39^+ ^or CD39^- ^Treg cells. Dot plots gated on CD4^+ ^T cells are shown with representative results. **(B) **Proliferative capacities of CD4^+^CD25^- ^responder alone and with autologous FACS-sorted CD4^+^CD25^+^CD39^+ ^Tregs or CD4^+^CD25^+^CD39^- ^Tregs subsets following anti CD3/anti-CD28 stimulation at the indicated S/R ratios on day 3. Statistical data are from three separate experiments with different donors. * *P *< 0.01, *vs*. the effects of CD39^+ ^Tregs at the corresponding S/R ratios.

CTLA-4, Ki-67, and HLA-DR are usually used as the phenotype markers for Treg cells. Thus, we further assessed the co-expression of CD39 and these molecules on FoxP3^+^CD4^+ ^T cells. Results revealed that the indicated markers expressed on CD39^+ ^Tregs were higher than those on the CD39^- ^Tregs (by ~1-fold) (Figure 2A), suggesting that CD39^+ ^Treg cells might be stronger suppressors than CD39^- ^Tregs. Functional assays indicated that CD39^+^Treg cells exhibited stronger suppressive ability to inhibit the proliferation of CD4^+^CD25^- ^T cells at different S/R ratios, as compared to the CD39^- ^Treg cells (Figure 2B). Furthermore, such suppressive effects demonstrated a dose-dependent activity because higher S/R ratio correlated with lower proliferation of the responder cells (Figure 2B).

In addition, we also observed that the CD39 molecule could be expressed on Th1 and Th17 cells, as indicated by the CD39int and CD39high groups displaying a small fraction of CD39^+^CD4^+ ^T cells capable of secreting IFN-γ and IL-17A, respectively (Additional file 2). This finding further suggested that CD39 might be capable of distinguishing Th1 or Th17 cells into different subpopulations with different functions.

Together, these data demonstrated that although CD39 expression appears to be highly variable on Treg cells, it can define a discrete subset of CD4^+^Foxp3^+ ^Treg cells, namely the CD39^+^Foxp3^+ ^Tregs. Since CD39 is preferentially expressed by Treg cells, the CD39^+ ^Treg cells were subjected to further investigations to determine their clinical significance in HBV infection.

### CD39^+^FoxP3^+ ^Treg populations correlate with hepatitis B progression

**Figure 3:**
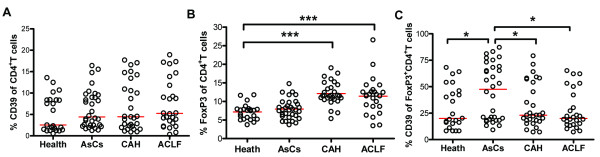
**CD39 expression on Treg cells in HBV disease progression**. Scatter plots summarizing flow cytometry data and showing the percentage of **(A) **CD4^+ ^T cells expressing CD39, **(B) **CD4^+ ^T cells expressing FoxP3, and **(C) **FoxP3^+^CD4^+ ^T cells expressing CD39. The horizontal lines of **(A) **and **(C) **indicate the medians, and the horizontal line of **(B) **indicates the mean value. * *P *< 0.05; *** *P *< 0.001.

The frequency of total CD39^+^CD4^+ ^T cells in AsCs, CAH and ACLF patients was examined. Results showed that there was no difference in the proportion of CD39 expression on CD4^+ ^T cells in AsCs, CAH and ACLF patients, as compared to the healthy control (Figure 3A). We further found that the frequency of total Treg cells (CD4^+^FoxP3^+ ^T cells) was significantly higher in CAH and ACLF patients than in normal controls (Figure 3B). However, the proportion of CD39-expressing Tregs was more dynamic. The CD39^+^Treg proportion was higher in AsCs patients than in healthy controls (*P *< 0.05). However, this proportion decreased significantly (nearly to the level in healthy controls) in the CAH and ACLF patients as compared with that in AsCs patients (*P *< 0.05, respectively) (Figure 3C). Thus, distinction of Treg cell subsets based on the combination of CD39 and FoxP3 expression is a highly informative approach for assessing the dynamics of Treg cell differentiation under HBV disease conditions.

### Circulating CD39^+ ^Treg cell level is correlated with HBV copy number and ALT level, but not with HBeAg expression

**Figure 4:**
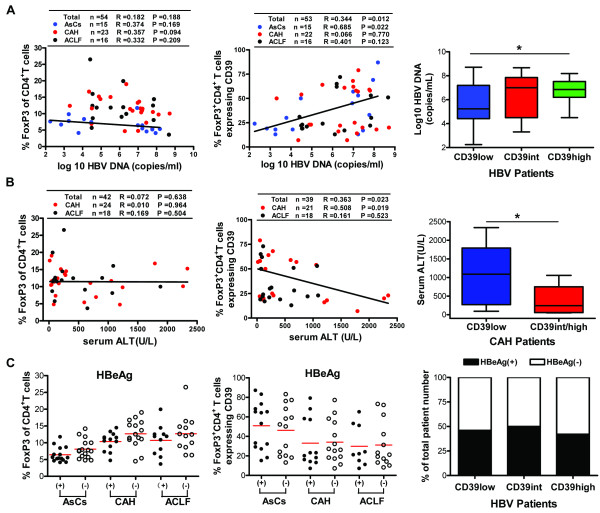
**The frequency of CD39^+ ^Treg cells is correlated with HBV copy number and ALT level, but not HBeAg expression**. (A) Correlations are shown between serum HBV loads and total Tregs (left panel) or CD39^+ ^Tregs (middle panel) in AsCs, CAH and ACLF patients. The HBV copies in CD39high/int/low groups are summarized in CD4^+ ^T cells expressing different levels of CD39 molecule (right panel). **(B) **Correlations are shown between serum ALT levels and total Tregs (left panel) or CD39^+ ^Tregs (middle panel) in CAH and ACLF patients. The ALT levels in CD39high/int/low groups are summarized in CD4^+ ^T cells expressing different levels of CD39 molecule (right panel). **(C) **Scatter plot summarizing flow cytometry data shows the percentage of CD4^+ ^T cells expressing FoxP3 (left panel) or CD4^+^FoxP3^+ ^T cells expressing CD39 (middle panel) in HBeAg(+)/(-) hepatitis B patients. The percentages of HBeAg(+) or (-) patients of the total patient number in CD39high/int/low groups are shown in the right panel. Mean values are indicated by the horizontal line. Bars represent mean ± SD. R is the correlation coefficient, and *P*-values are shown. * *P *< 0.05.

The body of published research to date has yet to consistently demonstrate whether circulating CD4^+^CD25^+ ^Treg frequency is correlated with HBV replication [3]. Therefore, we analyzed the correlation between Treg subsets and HBV copy number by measuring CD39 expression, serum ALT levels, HBV DNA load, and HBeAg in AsCs, CHB and ACLF patients, and comparing the results with those from total FoxP3^+ ^Treg cells. Results showed that there were no significant correlations between total FoxP3^+ ^Tregs and HBV viral copies (r = 0.182, *P *= 0.188) (Figure 4A, left panel). In contrast, when the frequency of Treg cells was analyzed on the basis of CD39 expression, it was significantly and positively correlated with the collective serum HBV viral copies in all tested hepatitis B patients (n = 53, r = 0.344, *P *= 0.012); however, in concrete disease status, significant correlation was only observed in AsCs patients (*r *= 0.685, *P *= 0.022) (Figure 4A, middle panel). Moreover, when analysis was performed with the CD39high/int/low groups, the HBV viral copies were found to be significantly increased in the CD39high group, as compared with the CD39low group in all HBV-infected subjects (*P *< 0.05) (Figure 4A, right panel).

Next, the correlation between Treg subsets was analyzed based on CD39 and serum ALT. Similar with the results described above for the HBV viral copies, there were no significant correlations found between total FoxP3^+ ^Tregs and serum ALT (*r *= 0.072, *P *= 0.638) (Figure 4B, left panel). In contrast, the frequency of CD39^+^FoxP3^+ ^Treg cells was significantly negatively correlated with serum ALT in total hepatitis B patients (n = 39, r = 0.344, *P *= 0.012), and a strong correlation was also observed in CAH patients (r = 0.508, *P *= 0.019) (Figure 4B, middle panel). Further analysis indicated that the serum ALT levels were increased in the CD39low group, as compared with the CD39int/high groups in CAH patients (*P *< 0.05), but not in the ACLF patients (Figure 4B, right panel).

In addition, we observed that there were no marked differences in the frequencies of total FoxP3^+ ^Tregs or CD39^+^FoxP3^+ ^Tregs between hepatitis B patients with or without serum HBeAg expression (Figure 4C, left and middle panels). Similar results were observed in the CD39high/int/low groups (Figure 4C, right panel).

### CD39^+ ^Treg cells accumulate in the livers of CHB patients

**Figure 5:**
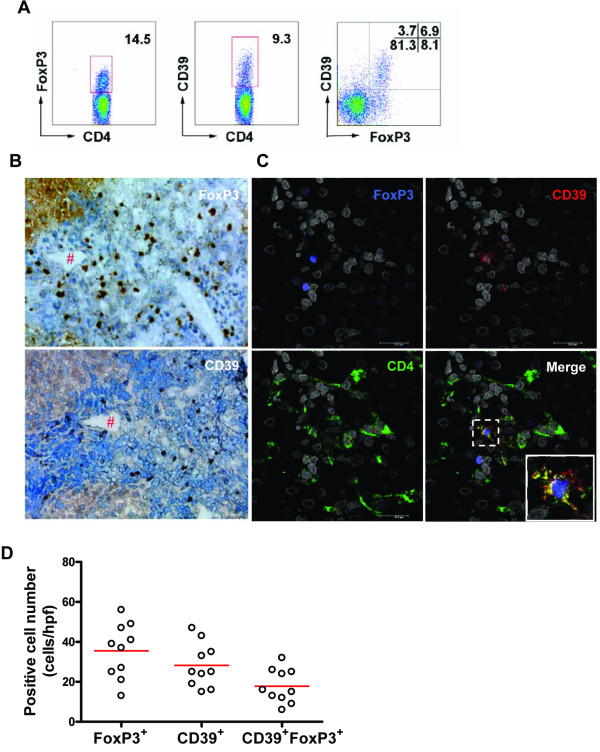
**Liver infiltration of CD39^+ ^Treg cells in hepatitis B patients**. (A) LILs from distal liver tissue of patients with liver hemangioma and HBV infection (HAI: S3/G3) were stained with anti-CD4, -CD45RA, -FoxP3, or -CD39 mAbs and analyzed by FCM assays. **(B) **Diagnostic needle-biopsy liver samples of CHB patients were stained with anti-CD39 or -FoxP3 mAb. # denotes the same region of adjacent sections. **(C) **Co-localization of CD4, CD39, and FoxP3 expressions are shown by immunofluorescence in diagnostic needle-biopsy liver samples of CHB patients. **(D) **Numbers of CD39^+^FoxP3^- ^/CD39^-^FoxP3^+ ^/CD39^+^FoxP3^+ ^cells in liver portal areas are shown in CAH patients. Each dot represents one individual. Horizontal bars represent the median positive numbers.

Next, the distribution of CD39^+ ^Treg cells was examined in HBV-infected livers by FCM analysis and morphological observation. The LILs of distal liver tissue (HAI: G3/S3) from the liver hemangioma patients with HBV infection were isolated and stained with various antibodies. As shown in Figure 5A, the CD39^+ ^Treg subset was detected in liver tissue by FCM assays. The CD39-expressing Foxp3^+ ^Tregs accounted for about 46% of the total Foxp3^+ ^Tregs, similar to our previous observations. We further examined the *in situ *distribution of the CD39^+ ^Treg subset in the livers of CHB patients by immunohistochemical and immunofluorescence staining techniques. Results showed that the CD39^+ ^and Foxp3^+ ^cells were mainly localized near the hepatic portal tract (Figure 5B). Using triple fluorescence staining, we confirmed that CD39 and FoxP3 expressions co-localized within regions with partial CD4^+ ^T cells (Figure 5C), indicating that the CD4^+^FoxP3^+^CD39^+ ^Treg cells did exist in the portal area of liver tissue and might be involved in hepatitis B pathogenesis. Quantitative analysis of the positive cells per hpf in liver portal areas in CAH patients is shown in Figure 5D. Results demonstrated that about 50% of total Foxp3^+ ^Tregs expressed CD39, in agreement with the observations from the above-described FACS assay.

## Discussion

While it is well-established that Treg cells play a central role in maintaining immune system balance, the roles of Treg cells in HBV disease have been largely inconclusive [30]. Inconsistent study designs and suboptimal definition of Tregs, such as relying solely on co-expression of CD4^+ ^and CD25^hi^Foxp3^+ ^to identify this subset, are the most likely culprits. In this study, we employed the CD39/ENTPD1, a newly described ectoenzyme that mediates regulatory T cell functions, to define the discrete subsets in Foxp3^+^CD4^+ ^Treg cells. We first characterized the peripheral and intrahepatic CD39^+^FoxP3^+^CD4^+ ^T cells (CD39^+ ^Tregs), most of which were found to be CD45RA^- ^Treg cells and displayed stronger suppressive effects than CD39^- ^Tregs. Then, we exploited this approach to investigate the role of Tregs in HBV infection and demonstrated, for the first time, that the CD39^+ ^Tregs are closely correlated to serum HBV copies and ALT, but not correlated with HBeAg in CHB patients.

In this study, FACS analysis of a large number of patient samples revealed a discrete distribution of CD39 expressing CD4^+ ^T cells in healthy controls and hepatitis B patients, which were discernable by their levels of CD39 expression. Accordingly, the CD39^+^CD4^+^T cells were categorized among three groups: CD39low, CD39int, and CD39high. Furthermore, the three groups showed distinctive association patterns with hepatitis disease status. Although we also observed an association between the frequency of total CD39^+ ^Treg cells with disease status, the subtypes of CD39^+^CD4^+ ^T cells provided more detailed information than the total CD39^+^CD4^+ ^T cells alone. For instance, when analysis was performed with the CD39high/int/low groups, the HBV viral copies were found to be significantly increased in the CD39high group, as compared with the CD39low group in all HBV-infected subjects; however, the serum ALT levels were increased in the CD39low group, as compared with the CD39int/high groups in CAH patients.

Friedman and colleagues found that tag-single nucleotide polymorphisms (SNPs) of the rs10748643 genotype correlated strongly with CD39 expression, whereby GG was associated with much higher levels than AA (46-77% increase) and AG carriers displayed intermediate expression levels in cell lines from subjects with Caucasian/European, Yoruban, Chinese, and Japanese ancestry [31]. In our study, we also observed a similar phenomenon. However, further investigations are required to determine whether the CD39 gene SNP is associated with any of the three groups (CD39low, CD39int, and CD39high) in this study. Such research is anticipated to provide further evidence for CD39 as a more suitable marker for Treg cells in combination with Foxp3.

To date, research has not been able to establish whether circulating CD4^+^CD25^+ ^Treg frequency is correlated with HBV replication. By using CD39 to define the conventional Treg cells, we found in this study that an increase in the proportion of CD39^+ ^Tregs occurred in AsCs patients; however, such an increase did not occur in the total Treg cells, which was similar to the proportions observed in healthy controls. Such a scenario demonstrated that CD39^+ ^Tregs may suppress the HBV-specific CD8^+ ^CTL effector function through the CD39/adenosine pathway [32], thereby maintaining immune tolerance to the invasive pathogen. This type of immunomodulation would be expected to further contribute to HBV replication in AsCs patients, who are characterized by high HBV load and low or normal serum levels [33,34].

Nikolova and colleagues reported that the Treg-CD39 subset inhibits cytokine production by HIV-specific CD8^+ ^T cells, an effect which could be partially relieved by pre-incubation of CD39^+ ^Tregs with anti-CD39 mAb [13]. Thus, we further analyzed the relationship between the proportion of CD39^+ ^Tregs and clinical pathological parameters. Statistical analysis showed that the percentage of CD39^+ ^Tregs was positively correlated with serum HBV load, especially in AsCs patients. However, we did not find any such correlation between HBV load and total Tregs. Based on the data in this study, there is no significant correlation between HBeAg expression and the frequency of total Tregs or CD39^+ ^Tregs. It is possible that the data in our study, which was obtained from routine clinical tests, did not represent an absolute quantification of the HBeAg protein expressions. Therefore, we were limited in our ability to investigate the relationship between Treg frequency and HBeAg expression in-depth. Our follow-up studies have been designed to address this issue while evaluating the potential clinical utility of manipulating CD39^+ ^Treg-mediated immunosuppression.

It is widely believed that the stages of CAH and ACLF in CHB infection are characterized by lower HBV copy number and higher serum ALT level, the latter of which often serves as a reliable marker of liver injury. Interestingly, in this study, we found that an increase in total Tregs was only observed in CAH and ACLF patients, as compared with AsCs patients or healthy controls. However, the significantly increased frequency of total Tregs showed no correlation with the increased serum ALT levels in these patients, thereby indicating that the increment of the total Tregs might reflect a homeostatic compensatory mechanism against inflammation. Nevertheless, the proportion of CD39^+ ^Tregs was found to be decreased in CAH and ACLF patients, as compared to those in the AsCs patients. We further observed that the percentage of CD39^+ ^Tregs was negatively correlated with serum ALT levels, especially in CAH patients, which might be due to subtle shifts in the subset composition of the total Tregs and which would be most clearly evidenced by the reduced frequency of CD39^+ ^Tregs.

It has been speculated that CD39 expression may be modulated by the inflammatory milieu. In some cases, this may lead to FoxP3 dysfunction and down-regulation of functional markers, such as CD39. Such impaired CD39^+ ^Treg cells might lose their ability to effectively hydrolyze extracellular ATP and ADP that is released by injured cells, thereby also losing their ability to inhibit the production of IL-17 [35] or other proinflammatory cytokines, such as IL-1β [33,36]. In a study of hepatic flares in chronic hepatitis B conducted by Tan *et al*., no increases in CD25^+^FoxP3^+^CD4^+ ^T cells or CD39^+^CD4^+ ^T cells were observed at any of the time points tested, suggesting that these cells may not play any role in inhibiting HBV-specific T cell responses *in vivo *[37]. However, as many non-Treg CD4^+ ^T cells also express CD39 molecules, it is possible that the role of the CD39^+ ^Treg subset was masked. Friedman and colleagues observed that, under conditions of inflammatory bowel disease, CD39-null mice suffer more severe injury than wild-type mice and exhibit a general tendency toward exaggerated Th1/IFN-γ responses [31]. Fletcher and colleagues reported that CD39^+^Foxp3^+ ^Treg cells can suppress pathogenic Th17 cells and are impaired in cases of multiple sclerosis [19]. Collectively, these evidences support the notion that while CD39^+ ^Treg cells can mediate strong inhibitive effects on other immune effector cells *in vivo*, the CD39^+ ^Tregs themselves can be modulated by various inflammatory factors. Loss of CD39 expression and of the related immunosuppresive functions during an inflammatory flare in CAH and ACLF patients could explain the negative correlation observed between the increased ALT levels and decreased CD39^+ ^Tregs frequency.

## Conclusions

In this study, we investigated the phenotype and the suppressive function of the newly identified CD39^+ ^Tregs in details. In HBV infected cases, the CD39^+ ^Tregs proportion was found to be increased in AsCs patients, but decreased in CAH and ACLF patients. Importantly, the CD39^+ ^Treg proportion is positively correlated with HBV load but inversely correlated with serum ALT level. These findings indicate that CD39 expression on FoxP3^+ ^Treg cells is involved in disease progression of HBV infection, suggesting that CD39^+ ^Treg populations are dynamic and can be followed longitudinally, thereby opening a new avenue towards therapeutic interventions aimed at modulating Treg functions.

## Authors' contributions

Yan Tang carried out the FCM and immunohistochemistry analysis. Li Jiang contributed to collecting the clinic data of CHB patients. Yanhua Zheng carried out the immunofluorescence assay and the FCM analysis. Yuzhang Wu and Bing Ni conceived the study, and participated in its design and coordination and drafted the manuscript equally. All authors read and approved the final manuscript.

## Supplementary Material

Additional file 1 **Figure S1 Subdivision of CD4^+ ^T cells expressing CD39 molecules**. According to the above distribution patterns of the scatter diagram for the frequency of CD4^+ ^T cells expressing CD39 in healthy controls and hepatitis B patients in our study, the total cohort were subdivided into three groups according to the frequency of CD39-expressing CD4^+ ^T cells: CD39low (< 5%), CD39int (5 ~ 10%), and CD39high (> 10%). The dotted lines indicate the boundaries between each group. Click here for file

Additional file 2 **Figure S2 CD39^+ ^CD4^+ ^T cells may express proinflammatory cytokines**. PBMCs from healthy donors were stained with anti-CD4, -CD39, -IFN-γ, or -IL-17A mAbs and analyzed by FACS. Dot plots gated on CD4^+ ^T cells are shown from a representative sample. Click here for file
