# Supplementary figures and images for: Expression of CD39 on FoxP3+ T regulatory cells correlates with progression of HBV infection
Source: BMC Immunol. 2012 Apr 11;13:17. doi: 10.1186/1471-2172-13-17 (PMC3364870; doi:10.1186/1471-2172-13-17)

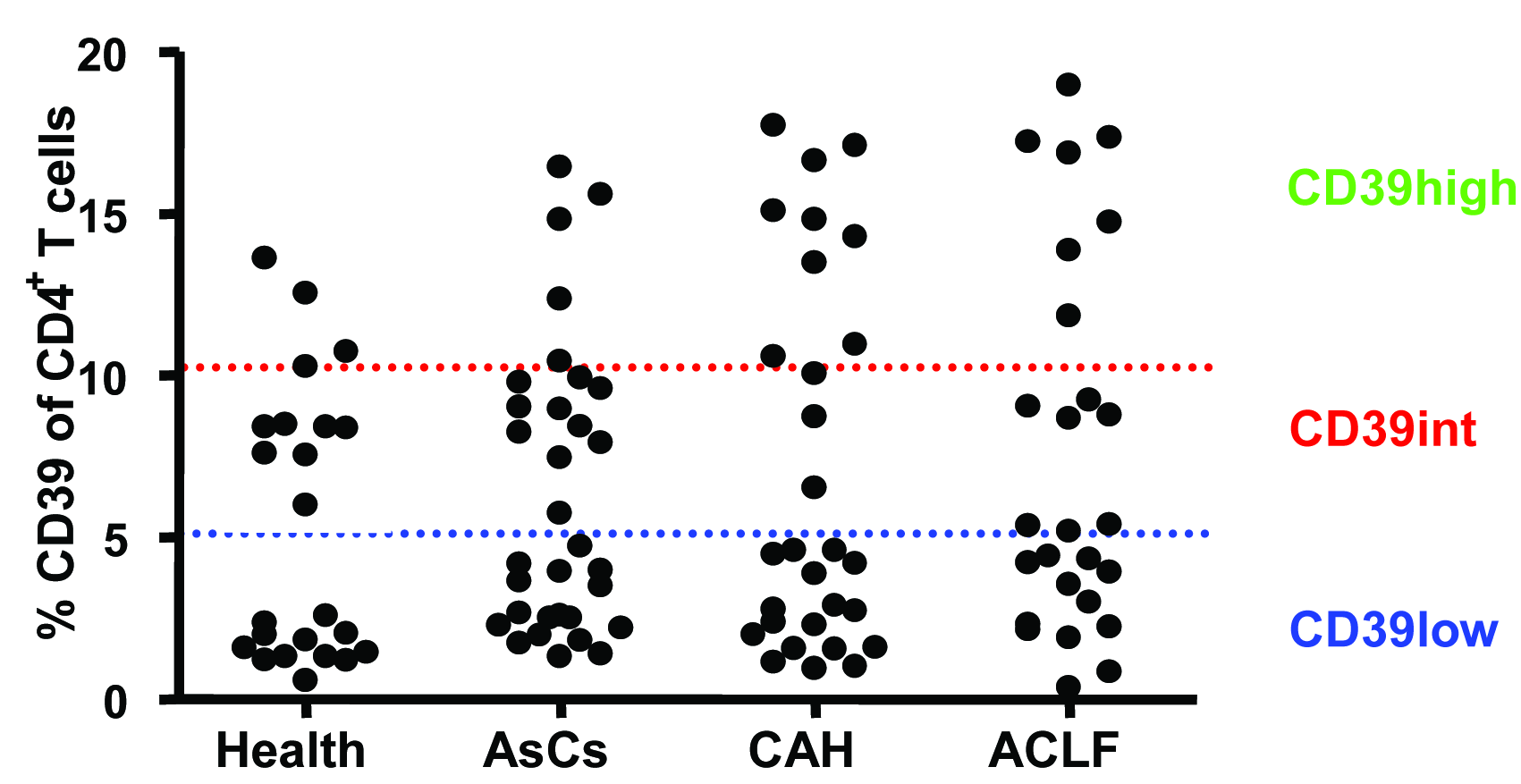

Supplement: Additional file 1 — Figure S1 Subdivision of CD4+ T cells expressing CD39 molecules. According to the above distribution patterns of the scatter diagram for the frequency of CD4+ T cells expressing CD39 in healthy controls and hepatitis B patients in our study, the total cohort were subdivided into three groups according to the frequency of CD39-expressing CD4+ T cells: CD39low (< 5%), CD39int (5 ~ 10%), and CD39high (> 10%). The dotted lines indicate the boundaries between each group. [file 1471-2172-13-17-S1.TIFF]

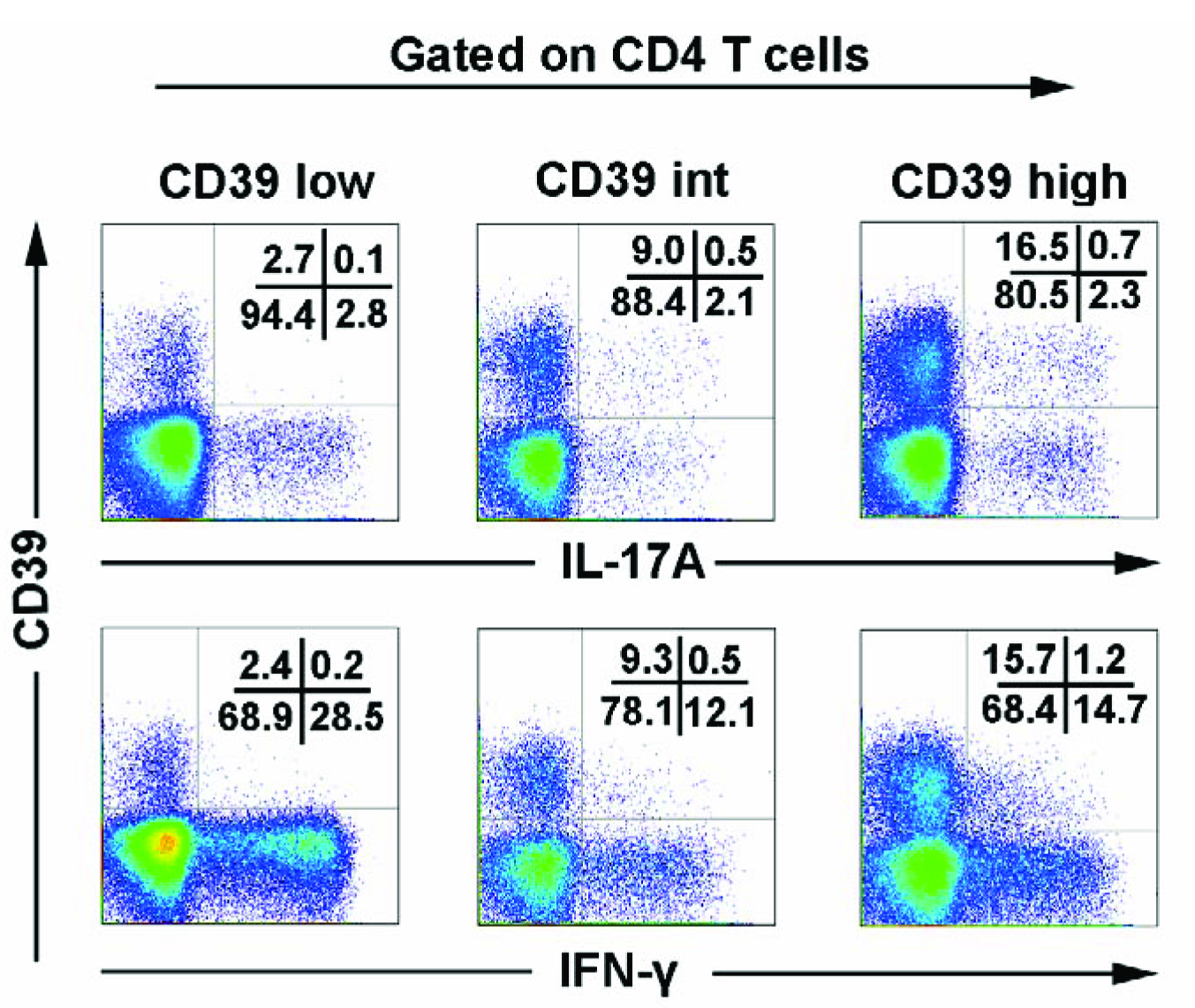

Supplement: Additional file 2 — Figure S2 CD39+ CD4+ T cells may express proinflammatory cytokines. PBMCs from healthy donors were stained with anti-CD4, -CD39, -IFN-γ, or -IL-17A mAbs and analyzed by FACS. Dot plots gated on CD4+ T cells are shown from a representative sample. [file 1471-2172-13-17-S2.TIFF]
